# Supplementary material for: Telephone-Based Coaching and Prompting for Physical Activity: Short- and Long-Term Findings of a Randomized Controlled Trial (Movingcall)
Source: Int J Environ Res Public Health. 2019 Jul 23;16(14):2626. doi: 10.3390/ijerph16142626 (PMC6678542; doi:10.3390/ijerph16142626)
Supplement: Supplementary file 1 [file ijerph-16-02626-s001.zip › ijerph-558236-supplementary/S1 Supplementary file on perceived fitness.pdf]

International Journal of
Environmental Research and Public Health

MDPI

Supplementary Material

Telephone-Based Coaching and Prompting for Physical Activity: Short- and Long-Term Findings of a Randomized Controlled Trial (Movingcall)

Xenia Fischer, Jan-Niklas Kreppke, Lukas Zahner, Markus Gerber, Oliver Faude and Lars Donath

S1: Supplementary File on Perceived Fitness

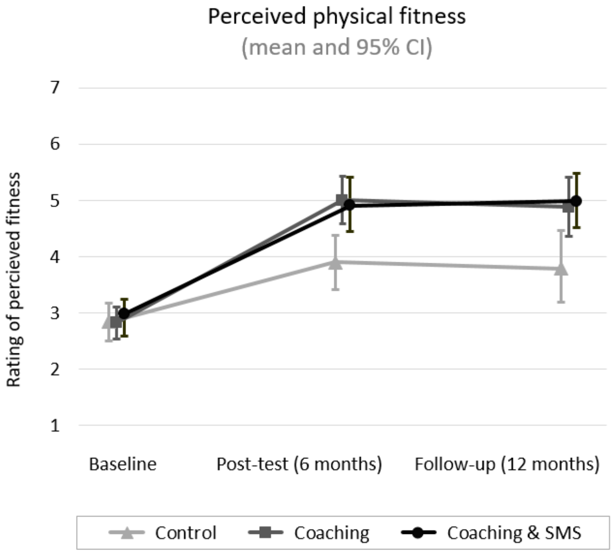

Figure 1. Unadjusted means and boot-strapped bias-corrected and accelerated confidence intervals of self-rated physical fitness. *N* = 285 at baseline, *n* = 205 at 6 months and *n* = 177 at 12 months.

Table 1. Adjusted changes in perceived physical fitness within groups and differences between groups at each time point

	Adjusted Mean Change from Baseline (95% CI)			Pairwise Comparison: Differences among Groups in Change from Baseline (95% CI)		
**M**	**Control**	**Coaching**	**Coaching and SMS**	**Coaching vs. Control**	**Coaching and SMS vs. Control**	**Coaching and SMS vs. Coaching**
6	1.1<br>(0.7 to 1.5)	2.1<br>(1.7 to 2.5)	2.0<br>(1.5 to 2.4)	1.0<br>(0.4 to 1.6)	0.9<br>(0.3 to 1.5)	-.1<br>(-0.7 to 0.4)
12	0.9<br>(0.4 to 1.4)	2.1<br>(1.7 to 2.5)	2.1<br>(1.7 to 2.5)	1.2<br>(0.5 to 1.8)	1.2<br>(.6 to 1.8)	0.04<br>(-.5 to 0.6)

M = Months.

© 2019 by the authors. Submitted for possible open access publication under the terms and conditions of the Creative Commons Attribution (CC BY) license (http://creativecommons.org/licenses/by/4.0/).
