# Supplementary material for: Telephone-Based Coaching and Prompting for Physical Activity: Short- and Long-Term Findings of a Randomized Controlled Trial (Movingcall)
Source: Int J Environ Res Public Health. 2019 Jul 23;16(14):2626. doi: 10.3390/ijerph16142626 (PMC6678542; doi:10.3390/ijerph16142626)
Supplement: Supplementary file 1 [file ijerph-16-02626-s001.zip › ijerph-558236-supplementary/S2 Supplementary file on acceptance and perception related questions.pdf]

International Journal of
Environmental Research and Public Health

MDPI

Supplementary Material

Telephone-Based Coaching and Prompting for Physical Activity: Short- and Long-Term Findings of a Randomized Controlled Trial (Movingcall)

Xenia Fischer, Jan-Niklas Kreppke, Lukas Zahner, Markus Gerber, Oliver Faude and Lars Donath

S2: Supplementary File on Acceptance and Perception Related Questions

Table1. Acceptance and perception related questions: Mean and standard deviation per study arm.

				**Control**	**Coaching**	**Coaching and SMS**		
	**Time of Assessment**	**Translated Question**	**Translated Answer on Likert Scale**	**Mean (SD)**	**Mean (SD)**	**Mean (SD)**	**p-value ANOVA**	**η2**
**General acceptance and perception of intervention**	6 months	In general, I am satisfied with this intervention.	1 = not true<br>2 = rather not true<br>3 = rather true<br>4 = true	**2.0 (0.8)**	**3.5 (0.6)**	**3.7 (0.6)**	< 0.001	0.481
	6 months	The program helps me to achieve my physical activity and exercise goals.	1 = not true<br>2 = rather not true<br>3 = rather true<br>4 = true	**1.7 (0.8)**	**3.4 (0.7)**	**3.4 (0.6)**	< 0.001	0.463
	6 months	This program helps me to be more physical active in my everyday life in the long-term (>1 year).	1 = not true<br>2 = rather not true<br>3 = rather true<br>4 = true	**2.2 (1.1)**	**3.5 (0.8)**	**3.6 (0.6)**	< 0.001	0.284

	6 months	This program will help me in the future to motivate myself to be regularly physically active.	1 = not true<br>2 = rather not true<br>3 = rather true<br>4 = true	**2.1 (1.1)**	**3.4 (0.7)**	**3.4 (0.7)**	< 0.001	0.286
**Activity profile**	3 months	In general, the profile on www.movingcall.com is understandable and easy to use.	1 = not true<br>2 = rather not true<br>3 = rather true<br>4 = true	**2.8 (1.0)**	**3.3 (0.7)**	**3.3 (0.7)**	< 0.001	0.074
	3 months	Did you use "my plan"? (e.g. to adapt the plan, to look up exercises in the catalogue or to write a note)	1 = No, never<br>2 = Rarely<br>3 = Yes, but irregularly<br>4 = Yes, regularly	**2.6 (0.9)**	**3.3 (0.8)**	**3.3 (0.8)**	< 0.001	0.090
**Perception and participation of the control group**	3 months	How often did you read the entire recommendation on physical activity?	Number	**2.4 (3.0)**				
	3 months	Is the recommendation formulated in an understandable way?	1 = no<br>2 = rather no<br>3 = rather yes<br>4 = yes	**3.6 (0.8)**				
	3 months	Is the advice in your recommendation tailored to your personal situation in life?	1 = no<br>2 = rather no<br>3 = rather yes<br>4 = yes	**2.6 (0.9)**				

**Perception and participation of the coaching and the coaching & SMS group**	6 months	Did the phone calls take place regularly?	1 = no<br>2 = mostly<br>3 = yes	**2.9 (0.3)**	**2.9 (0.3)**	0.520	0.009
	6 months	The duration of the phone calls was …	1 = too short<br>2 = appropriate<br>3 = too long	**2.0 (0.2)**	**2.0 (0.1)**	0.368	0.005
	6 months	The intervals of the phone calls were …	1 = too short<br>2 = appropriate<br>3 = too long	**2.0 (0.3)**	**2.0 (0.2)**	0.749	0.004
	6 months	My coach and I had a trusting relationship.	1 = not true<br>2 = rather not true<br>3 = rather true<br>4 = true	**3.8 (0.5)**	**3.9 (0.3)**	0.524	0.015
	6 months	Our relationship was characterized by mutual respect.	1 = not true<br>2 = rather not true<br>3 = rather true<br>4 = true	**3.9 (0.2)**	**4.0 (0.2)**	0.311	0.006
	6 months	I was coached according to my current state of my physical activity (e.g. appropriate goals were set).	1 = not true<br>2 = rather not true<br>3 = rather true<br>4 = true	**3.9 (0.4)**	**3.9 (0.4)**	0.498	0.009
	6 months	I do collaborate with my coach during the coaching process.	1 = not true<br>2 = rather not true<br>3 = rather true<br>4 = true	**3.7 (0.5)**	**3.8 (0.5)**	0.649	0.006

**Perception and participation of the coaching & SMS group**	6 months	Did you always receive 4 SMS between the calls?	1 = I have not received any SMS<br>2 = No, not always<br>3 = Yes, always	**2.9 (0.3)**
	6 months	Do you perceive the SMS overall as supporting?	1 = No, they are not supportive<br>2 = No, they are rather not supportive<br>3 = Yes, they are more supportive<br>4 = Yes, they are supportive	**3.4 (0.8)**
	6 months	How do you rate the frequency of the SMS?	1 = Too rare<br>2 = Appropriate<br>3 = Too often	**2.1 (0.4)**

© 2019 by the authors. Submitted for possible open access publication under the terms and conditions of the Creative Commons Attribution (CC BY) license (http://creativecommons.org/licenses/by/4.0/).
